# Supplementary material for: Using Ipomoea aquatic as an environmental-friendly alternative to Elodea nuttallii for the aquaculture of Chinese mitten crab
Source: PeerJ. 2019 Apr 19;7:e6785. doi: 10.7717/peerj.6785 (PMC6476289; doi:10.7717/peerj.6785)
Supplement: Supplemental Information 1 — DO concentrations measured in preliminary research in 2016. E. nuttallii, Elodea nuttallii; O. sativa, Oryza sativa; I. aquatic, Ipomoea aquatica. Different small letters on the box represent significant differences between treatments at 0.05 levels (Tukey HSD). [file peerj-07-6785-s001.docx]

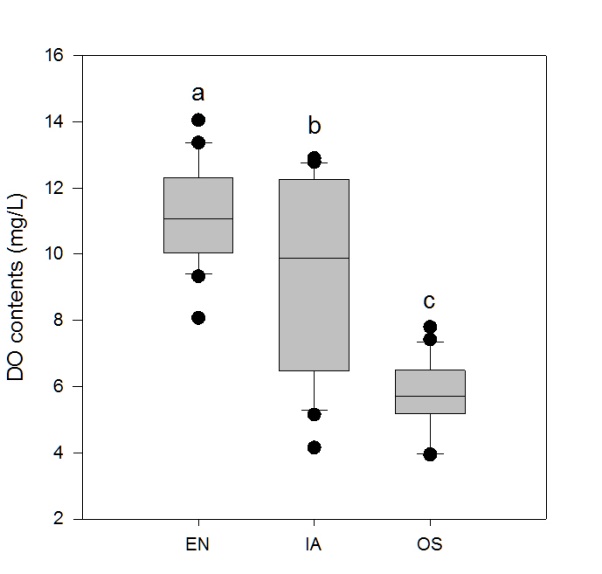


Fig. S1. DO concentrations measured in preliminary research in 2016. EN, *Elodea nuttallii*; OS, *Oryza sativa*; IA, *Ipomoea aquatica*. Different small letters on the box represent significant differences between treatments at 0.05 levels (Tukey HSD).
