# Supplementary material for: Using Ipomoea aquatic as an environmental-friendly alternative to Elodea nuttallii for the aquaculture of Chinese mitten crab
Source: PeerJ. 2019 Apr 19;7:e6785. doi: 10.7717/peerj.6785 (PMC6476289; doi:10.7717/peerj.6785)
Supplement: Supplemental Information 2 — Figure S2. Planform of rearing ponds in this study. The frame is consisted of rope and plastic balls. The plants that grow out of frames would be cut. Figure S3. The demo graph of E. nuttallii (Elodea nuttallii) trimming. The frame is consisted of rope and plastic balls. The stems and leaves of E. nuttallii that grow out of frames were trimmed manually using sickle, and just short stems (less than 1–2 cm) and roots of E. nuttallii were left. [file peerj-07-6785-s002.docx]

Trimming in the OS (*Oryza sativa*) treatment was not needed, because OS only grew in the frames, which occupied 60% area of total surface water in ponds. Trimming in the IA (*Ipomoea aquatica*) treatment was easy, because we can manually trim the stems and leaves of IA using sickle that grew out of the frames (Fig. S2). Trimming in the EN (*Elodea nuttallii*) treatment should be careful, because EN grew in the sediment. Local farmers generally cut the stems and leaves of EN, and short stems and roots were left for future growing. We trimmed EN as same as what farmers did (Fig. S3), and the left area of EN was limited to the 60% area of the total surface water area using the floating frames that consisted of rope and plastic balls.


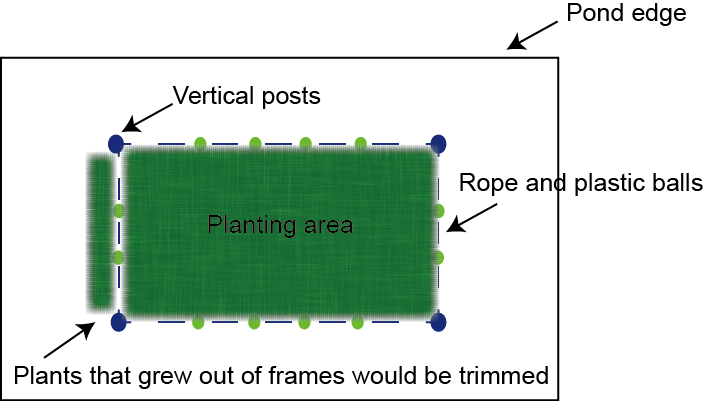


Fig. S2. Planform of rearing ponds in this study. The frame is consisted of rope and plastic balls. The plants that grow out of frames would be cut.


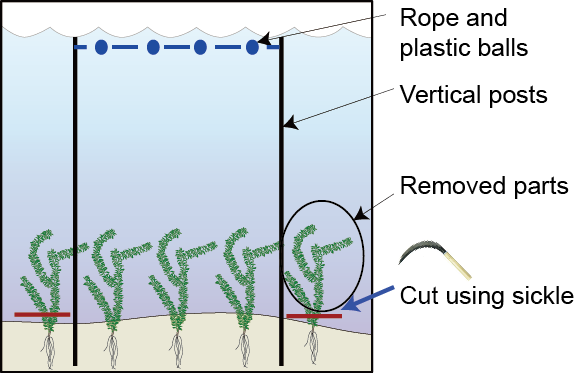


Fig. S3. The demo graph of EN (*Elodea nuttallii*) trimming. The frame is consisted of rope and plastic balls. The stems and leaves of EN that grow out of frames were trimmed manually using sickle, and just short stems (less than 1–2 cm) and roots of EN were left.
