# Supplementary material for: Using Ipomoea aquatic as an environmental-friendly alternative to Elodea nuttallii for the aquaculture of Chinese mitten crab
Source: PeerJ. 2019 Apr 19;7:e6785. doi: 10.7717/peerj.6785 (PMC6476289; doi:10.7717/peerj.6785)
Supplement: Supplemental Information 3 — The tissue-mixed plant samples that collected in different growth stages were used for nutrient concentration measurement. Nitrogen and phosphorus contents are determined by the Kjeldahl Nitrogen Determination method and the molybdate-ascorbic acid method, respectively. [file peerj-07-6785-s003.docx]

Table S1: Average nutrient concentrations of plants

| Plants | TN(%) | TP(%) |
| --- | --- | --- |
| *Ipomoea aquatic* | 1.28 | 0.08 |
| *Oryza sativa* | 4.13 | 0.28 |
| *Elodea nuttallii* | 1.45 | 0.46 |

Notes: The tissue-mixed plant samples that collected in different growth stages were used for nutrient concentration measurement. Nitrogen and phosphorus contents are determined by the Kjeldahl Nitrogen Determination method and the molybdate-ascorbic acid method, respectively.
