# Supplementary material for: Using Ipomoea aquatic as an environmental-friendly alternative to Elodea nuttallii for the aquaculture of Chinese mitten crab
Source: PeerJ. 2019 Apr 19;7:e6785. doi: 10.7717/peerj.6785 (PMC6476289; doi:10.7717/peerj.6785)
Supplement: Supplemental Information 4 — Different small letters in the same column represent significant differences among treatments. [file peerj-07-6785-s004.docx]

Table S2. Water nutrient concentrations in July 2.

| Treatments | TN (mg/L) | TP (mg/L) | NH_4_^+^-N (mg/L) | NO_3_^-^-N  (mg/L) | pH |
| --- | --- | --- | --- | --- | --- |
| EN | 0.73a | 0.13ab | 0.18a | 0.67a | 8.63a |
| IA | 0.59a | 0.14a | 0.15a | 0.43a | 7.61c |
| OS | 0.58a | 0.13b | 0.14a | 0.44a | 8.12b |

Different small letters in the same column represent significant differences among treatments.
