# Supplementary material for: Using Ipomoea aquatic as an environmental-friendly alternative to Elodea nuttallii for the aquaculture of Chinese mitten crab
Source: PeerJ. 2019 Apr 19;7:e6785. doi: 10.7717/peerj.6785 (PMC6476289; doi:10.7717/peerj.6785)
Supplement: Supplemental Information 5 — The denitrification and nitrification contribution prediction using PICRUSt1.1.3. [file peerj-07-6785-s005.docx]

The denitrification and nitrification contribution prediction using PICRUSt1.1.3.

The normalized_otus.biom file was generated using function “normalize_by_copy_number.py” with an otu table

#normalize_by_copy_number.py

# -i your_otu_table.biom

# -o normalized_otus.biom

# run the following scripts in PICRUSt1.1.3

# denitrification contribution ------------------------------------------------------------

metagenome_contributions.py -i normalized_otus.biom -l K00370, K00371, K00374, K02567, K02568, K00368, K15864, K04561, K02305, K00376, K00370, K00371, K10535, K10944, K10945, K10946 -o Denitrification_KO_metagenome_contributions.tab

# nitrification contribution --------------------------------------------------------------

metagenome_contributions.py -i normalized_otus.biom -l K00370, K00371, K00374, K02567, K02568, K00368, K15864, K04561, K02305, K00376, K00370, K00371, K10535, K10944, K10945, K10946 -o Nitrification_KO_metagenome_contributions.tab
